# Prognostic relevance of Centromere protein H expression in esophageal carcinoma

**DOI:** 10.1186/1471-2407-8-233

**Published:** 2008-08-13

**Authors:** Xian-Zhi Guo, Ge Zhang, Jun-Ye Wang, Wan-Li Liu, Fang Wang, Ju-Qin Dong, Li-Hua Xu, Jing-Yan Cao, Li-Bing Song, Mu-Sheng Zeng

**Affiliations:** 1 State Key Laboratory of Oncology in Southern China, Sun Yat-sen University Cancer Center, Guangzhou, PR China; 2 Department of Experimental Research, Sun Yat-sen University Cancer Center, Guangzhou, PR China; 3 Thoracic Carcinoma, Sun Yat-sen University Cancer Center, Guangzhou, PR China; 4 Pathology, Sun Yat-sen University Cancer Center, Guangzhou, PR China; 5 School of Pharmaceutical Sciences, Sun Yat-sen University, Guangzhou, PR China; 6 The Central Hospital of Xuhui District, Shanghai, PR China

## Abstract

**Background:**

Many kinetochore proteins have been shown to be associated with human cancers. The aim of the present study was to clarify the expression of Centromere protein H (CENP-H), one of the fundamental components of the human active kinetochore, in esophageal carcinoma and its correlation with clinicopathological features.

**Methods:**

We examined the expression of CENP-H in immortalized esophageal epithelial cells as well as in esophageal carcinoma cells, and in 12 cases of esophageal carcinoma tissues and the paired normal esophageal tissues by RT-PCR and Western blot analysis. In addition, we analyzed CENP-H protein expression in 177 clinicopathologically characterized esophageal carcinoma cases by immunohistochemistry. Statistical analyses were applied to test for prognostic and diagnostic associations.

**Results:**

The level of CENP-H mRNA and protein were higher in the immortalized cells, cancer cell lines and most cancer tissues than in normal control tissues. Immunohistochemistry showed that CENP-H was expressed in 127 of 171 ESCC cases (74.3%) and in 3 of 6 esophageal adenocarcinoma cases (50%). Statistical analysis of ESCC cases showed that there was a significant difference of CENP-H expression in patients categorized according to gender (*P *= 0.013), stage (*P *= 0.023) and T classification (*P *= 0.019). Patients with lower CENP-H expression had longer overall survival time than those with higher CENP-H expression. Multivariate analysis suggested that CENP-H expression was an independent prognostic marker for esophageal carcinoma patients. A prognostic value of CENP-H was also found in the subgroup of T3~T4 and N0 tumor classification.

**Conclusion:**

Our results suggest that CENP-H protein is a valuable marker of esophageal carcinoma progression. CENP-H might be used as a valuable prognostic marker for esophageal carcinoma patients.

## Background

During the proliferation of normal cells, the centrosome ensures the equal segregation of chromosomes to the postmitotic daughter cells by organizing the bipolar mitotic spindle. In contrast, in cancer cells multipolar mitotic spindles and various centrosomal anomalies, such as supernumerary centrosomes, centrosomes of abnormal size and shape, and prematurely split centrosomes are frequently observed [1-3] It is conceivable that such abnormalities disrupt normal chromosomal segregation, producing aneuploid cells and causing chromosomal instability (CIN).

CIN has been recognized as a hallmark of human cancer and is caused by continuous chromosome missegregation during mitosis[4]. Proper chromosome segregation requires a physical connection between spindle microtubules and centromeric DNA and this attachment occurs at proteinaceous structures called kinetochore [5]. Several kinetochore proteins have been identified in humans, and the list of kinetochore associated proteins continues to grow, including centromere protein (CENP)-A, CENP-B, CENP-C, CENP-E, CENP-F, CENP-H, CENP-I, and INCENP [6,7]. Studies have shown that kinetochore malfunction is a major cause of aneuploidy and is closely associated with CIN [8,9]. Normal expression of core kinetochore components is essential to prevent chromosome instability [10]. CENP-H was initially identified as a component of the mouse centromere [11]. Human CENP-H protein was recently isolated and shown to localize in the inner plate together with CENP-A and CENP-C and is a fundamental component of the active centromere complex [12,13]. Studies using budding yeast have shown that a molecular core consisting of CENP-A, CENP-C, CENP-H, and Ndc80/HEC plays a central role in linking centromeres to the spindle microtubule [14].

Recent research has shown that CENP-H is up-regulated in most colorectal cancers, and ectopic expression of CENP-H induces chromosome missegregation and aneuploidy in diploid cell lines [15]. Another study found significant association between the level of CENP-H mRNA expression and clinical stage in oral squamous cell carcinomas and indicated that human CENP-H was closely linked to the increased or abnormal cell proliferation in malignant conditions [16]. Previously, we have demonstrated a correlation between expression of CENP-H and both tumor progression and poor prognosis of the patients in the human nasopharyngeal carcinoma[17]. Although CENP-H may play an important role in chromosome instability and carcinogenesis, there are no reports on its role in tumorigenesis and progression of esophageal carcinoma. In this study, we thus investigated the CENP-H expression and its clinical significance in human esophageal carcinoma.

## Methods

### Cell lines

The NE-3 and 108CA cell lines were obtained from Dr. Jin (the University of Hong Kong, P. R. China) and were cultured in Keratinocyte-SFM (Invitrogen, Carlsbad, CA) supplemented with antibiotics (100 μg/μL streptomycin and 100 μg/μL penicillin). The NE-3 is an immortalized esophageal epithelial cell lines and the latter is an ESCC cell line[18,19]. The ESCC cell lines Eca-109, TE-1, and Kyse140 (Cell Bank of Type Culture Collection of Chinese Academy of Sciences, Shanghai, China) were grown in RPMI 1640 (Invitrogen) supplemented with 10% fetal bovine serum, 100 μg/μL streptomycin, and 100 μg/μL penicillin in a humidified incubator containing 5% CO2 at 37°C.

### Patients and tissue specimens

Twelve pairs of ESCC tissue specimens and corresponding nontumorous specimens were obtained from patients with ESCC who underwent surgical esophageal tissue resection at the Cancer Center of Sun Yat-sen University (Guangzhou, P. R. China) during 2007. Written informed consent was obtained from each patient before surgery. All excised samples were obtained within 1 h after the operation from tumor tissues and corresponding nontumorous tissues 5–10 cm from the tumor. All excised tissues were immediately placed in liquid nitrogen until further analysis. In addition, immunohistochemstry analysis was conducted on 177 paraffin-embedded samples, including 171 ESCC and 6 esophageal adenocarcinoma which were histologically and clinically diagnosed from the Cancer Center, Sun Yat-sen University, between 2001 and 2004. Prior to the use of these clinical materials for investigation, informed consent from patients and approval from the Institute Research Ethics Committee were obtained. Primary cancers of the esophagus were classified according to the pathological TNM classification [20]. Since the number of esophageal adenocarcinoma is small, clinical information of 171 ESCC samples is only described in detail in Table 1. Patients included 129 males and 42 females, of ages ranging from 33 to 82 years (mean, 56.7 years). The figures on metastasis pertain to its presence at any time in follow-up. The median follow-up time for overall survival was 25.0 months for patients still alive at the time of analysis, and ranged from 1 to 78 months. A total of 112 (65.5%) patients died during follow up.

**Table 1:** Clinicopathologic characteristics of patient samples and expression of CENP-H in esophageal carcinoma.

Characteristics	n(%)
	(n = 171)
Gender	
Male	129(75.4)
Female	42(24.6)
Age(y)	
≥ 60	103(60.2)
<60	68(39.8)
Stage	
I	10(5.8)
II a	75(43.9)
II b	14(8.2)
III	63(36.8)
IV	9(5.3)
Histological classification	
Squamous cell carcinoma	171(100.0)
Histological differentiation	
Well	55(32.2)
Moderate	72(42.1)
Poor	44(25.7)
Tumor diameter	
≥ 40 mm	72(42.1)
<40 mm	99(57.9)
Depth of invasion	
Submucosa	12(7.0)
Muscularis propria	58(33.9)
Adventitia	101(59.1)
pT classification	
T1	13(7.6)
T2	46(26.9)
T3	106(62.0)
T4	6(3.5)
pN classification	
YES	77(45.0)
NO	94(55.0)
pMetastasis	
YES	9(5.3)
NO	162(94.7)
Vital status(at follow-up)	
Alive	59(34.5)
Death because of esophageal carcinoma	109(63.7)
Death because of unknown cancer or other than esophageal carcinoma	3(1.8)
Expression of CENP-H	
Negative	44(25.7)
Positive	127(74.3)
Low expression	54(31.6)
High expression	73(42.7)

### RNA extraction and reverse transcription-PCR

Total RNAs from cells, tumor tissue and nontumorous tissues was extracted using Trizol reagent (Invitrogen) according to the manufacturer's instructions. The RNA was pretreated with DNase and used for cDNA synthesis with random hexamers. The full-length open reading frame of CENP-H was PCR amplified from cDNA samples of normal tissue and ESCC cell lines. The following primers were used for amplification of CENP-H: sense primer, 5'-TGCAAGAAAAGCAAATCGAA-3'; antisense primer, 5'-ATCCCAAGATTCCTGCTGTG-3'. Glyceraldehyde-3-phosphate dehydrogenase was amplified as an internal control using sense primer, 5'-AATCCCATCACCATCTTCCA-3' and antisense primer, 5'-CCTGCTTCACCACCTTCTTG-3'. The appropriate size of PCR products was confirmed by agarose gel electrophoresis.

### Protein extraction and immunoblotting

Frozen tissue samples were solubilized in lysis buffer [7 mol/L urea, 2 mol/L thiourea, 2% CHAPS, 0.1 mol/L DTT, 0.1% NP40, 40 mmol/L Tris-HCl] using a Polytron homogenizer following centrifugation (100,000 g) for 1 h at 4°C. Cultured cells were harvested in 1× SDS sample buffer [62.5 mmol/L Tris-HCl (pH 6.8), 2% SDS, 10% glycerol, and 5% 2-mercaptoethanol] and were heated for 5 min at 100°C. Protein concentration was determined by the Bradford assay (Bio-Rad Laboratories, Hercules, CA). Equal amounts of proteins were separated electrophoretically on 12% SDS polyacrylamide gels and transferred onto polyvinylidene difluoride membranes (Amersham Pharmacia Biotech, Piscataway, NJ). The membrane was probed with an anti-CENP-H rabbit polyclonal antibody (1:1,000; Bethyl Laboratories, Montgomery, TX). Expression of CENP-H was determined with horseradish peroxidase-conjugated anti-rabbit immunoglobulin G (1:3,000; Amersham Pharmacia Biotech) and enhanced chemiluminescence (Amersham Pharmacia Biotech) according to the manufacturer's suggested protocols. An anti-α-tubulin mouse monoclonal antibody (1:1,000; Santa Cruz Biotechnology, Santa Cruz, CA) was used to confirm equal loading.

### Immunohistochemistry

Immunohistochemistry was done to study altered protein expression in 177 human esophageal cancer tissues. In brief, paraffin-embedded specimens were cut into 4-μm sections and baked at 65°C for 30 min. The sections were deparaffinized with xylenes and rehydrated. Sections were submerged into EDTA antigenic retrieval buffer and microwaved for antigenic retrieval. The sections were treated with 3% hydrogen peroxide in methanol to quench the endogenous peroxidase activity, followed by incubation with 3% bovine serum albumin to block the nonspecific binding. Rabbit polyclonal anti-CENP-H (1:500; Bethyl Laboratories) was incubated with the sections overnight at 4°C. For negative controls, the primary antibody was replaced by normal rabbit serum. After washing, the tissue sections were treated with biotinylated anti-rabbit secondary antibody (Zymed, San Francisco, CA), followed by further incubation with streptavidin horseradish peroxidase complex (Zymed). The tissue sections were immersed in 3-amino-9-ethyl carbazole and counterstained with 10% Mayer's hematoxylin, dehydrated, and mounted in Crystal Mount. The degree of immunostaining of formalin-fixed, paraffin-embedded sections was reviewed and scored by two independent observers. The proportion of the stained cells and the extent of the staining were used as criteria of evaluation. For each case, at least 1,000 tumor cells were analyzed and the percentage of positively nuclear stained tumor cells was recorded. For each sample, the proportion of CENP-H-expressing cells varied from 0% to 100%, and the intensity of nuclear staining varied from weak to strong. One score was given according to the percent of positive cells as: < 5% of the cells:1 point; 6–35% of the cells:2 point; 36–70% of the cells:3 point; >71% of the cells: 4 point. Another score was given according to the intensity of staining as negative staining: 1 point; weak staining (light yellow): 2 point; moderate staining(yellowish brown): 3 point; and strong staining(brown): 4 point. A final score was then calculated by multiple the above two scores. If the final score was equal or bigger than four, the tumor was considered high expression; otherwise, the tumor was considered low expression [21].

### Statistical analysis

Since the number of esophageal adenocarcinoma is small, all statistical analyses were carried out in 171 ESCC cases using the SPSS 13.0 statistical software package. Mann-Whitney *U *test was used to analyze the relationship between CENP-H expression and clinicopathologic characteristics. Survival curves were plotted by the Kaplan-Meier method and compared by the log-rank test. The significance of various variables for survival was analyzed by the Cox proportional hazards model in the multivariate analysis. *P *< 0.05 in all cases was considered statistically significant.

## Results

### Expression of CENP-H in esophageal carcinoma cell lines

**Figure 1:**
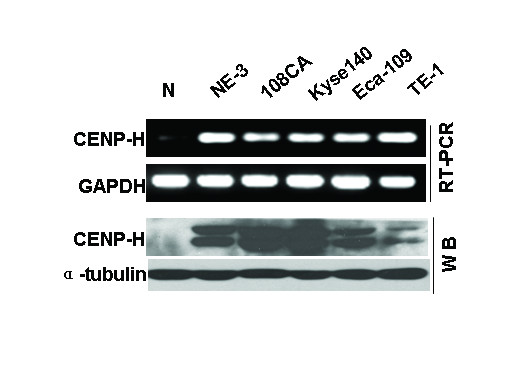
**Expression analysis of CENP-H mRNA and protein in an esophageal immortalization cell line (NE3) and 4 esophageal carcinoma cell lines 108CA, Kyse 140, Eca-109 and TE-1 by reverse transcription-PCR and Western blotting**. A normal esophageal tissue was used as a control.

To investigate the expression levels of CENP-H transcripts and protein in esophageal cancer cell lines, semiquantitative reverse transcription-PCR analysis and Western blotting analysis were done in NE-3, 108CA, Eca-109, TE-1, and Kyse140 cell lines. All five cell lines showed higher level expression of CENP-H mRNA in comparison with the normal esophageal tissue (Fig. 1). Western blotting analysis showed that CENP-H protein was highly expressed in all cell lines, whereas it was weakly detected in normal esophageal tissue (Fig. 1).

### Expression of CENP-H in paired esophageal cancer and nontumorous tissues

**Figure 2:**
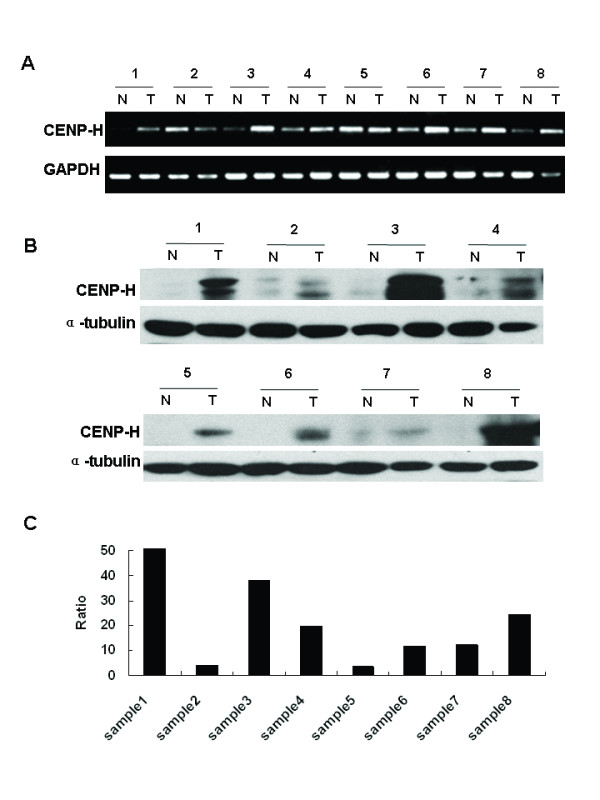
**Expression analysis of CENP-H mRNA and protein in normal esophageal tissues and esophageal carcinoma cancer tissues by reverse transcription PCR (A) and Western blots (B)**. A. Reverse transcription PCR results in 8 pairs of esophageal tissues. B. Western blots results in 8 pairs of esophageal tissues. (N means normal and T means tumor). C. The density ratio of western blots results by Quantity one.

As we detected CENP-H overexpression in esophageal carcinoma cell lines, we were interested in investigating the status of CENP-H expression in esophageal carcinoma biopsies. We initially did RT-PCR analysis on twelve esophageal tumor tissues (T) versus normal esophageal tissues (N) obtained from the same patients. The expression of GAPDH was examined as an internal control. As shown in Fig 2A, the expression levels of CENP-H mRNA in most cancer tissues (6/12) was higher than in normal tissues, and quantitative analysis showed that there was a significant difference in CENP-H expression between cancer tissues and normal tissues. To determine if the higher level of CENP-H mRNA expression revealed by RT-PCR analysis was directly linked to increased levels of CENP-H protein expression, we performed Western blot analysis with protein extracts from matched samples of tumor (T) and adjacent normal tissue (N) (from which the mRNA samples were extracted). As shown in Fig 2B, CENP-H was found to be greatly overexpressed in 8 of 12 cases of primary esophageal carcinoma, whereas only faint CENP-H expression was found in the normal esophageal tissues, with at least twofold overexpression of CENP-H in cancer tissues compared with normal tissues in these 8 cases (the density ratio was from 2.3270 to 51.735) (Fig 2C). There was no significant difference in other four pairs of esophageal carcinoma biopsies, which showed low expression of CENP-H in both normal and tumor tissues (Data not shown). Taken together, these data demonstrate that CENP-H is highly expressed at both mRNA and protein levels in most of the esophageal cancer tissues. However, the inconsistent expression of CENP-H at mRNA and protein levels for case 2 and 5 indicate that the deregulation of CENP-H could happen at either transcriptional or post-transcriptional stage.

### Expression of CENP-H in archival esophageal cancer tissues

**Figure 3:**
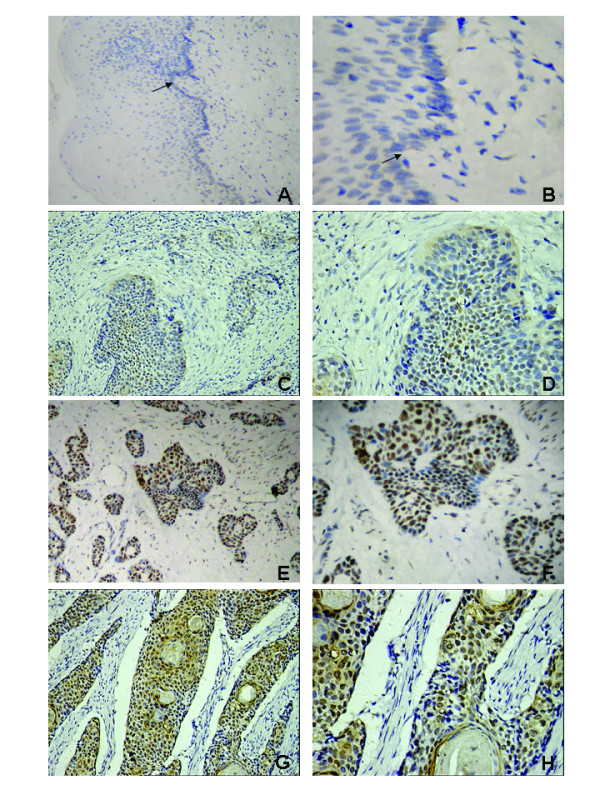
**Expression analysis of CENP-H protein by immunohistochemistry**. CENP-H expression was mainly localized within nuclei of tumor cells, and diffuse staining was observed in some tumor cells. CENP-H is not expressed in normal epithelial cells. A and B Staining of CENP-H in normal esophageal epithelial tissue (arrow, normal epithelial cells). C and D, low expression of CENP-H in esophageal carcinoma tissues (200 and 400, respectively). E, F, G and H, high expression of CENP-H in esophageal carcinoma tissues (200 and 400, respectively).

Expression and subcellular localization of CENP-H protein was determined by immunohistochemistry in 177 paraffin-embedded, archival esophageal cancer tissues. CENP-H protein was detected in 127 of 171 ESCC cases(74.3%) and in 3 of 6 esophageal adenocarcinoma cases (50%). The subcellular location of CENP-H was nuclei and cytoplasm of tumor cells, but mainly nuclei. In addition, diffuse staining was observed in some tumor cells (Fig. 3C to 3H). Fig. 3C and 3D showed low expression of CENP-H in esophageal carcinoma tissues, and Fig. 3E and 3F showed high expression of CENP-H located in nuclei in esophageal carcinoma tissues. Fig. 3G and 3H showed high expression of CENP-H located in mainly nuclei and partly cytoplasm in esophageal carcinoma tissues. No specific CENP-H staining was observed in normal esophageal epithelial cells (Fig. 3A and 3B) and in the surrounding stroma cells.

### Correlation between CENP-H protein expression and clinicopathological features

Table 2 shows the relationship between the expression of CENP-H protein and clinical characteristics in 171 ESCC cases. There was no significant correlation between the expression level of CENP-H protein and age, histological classification, histological differentiation, tumor diameter, depth of invasion, pN classification or distant metastasis of esophageal cancer patients. However, the expression of CENP-H is closely associated with stage of esophageal cancer patients (*P *= 0.023) and T classification (*P *= 0.019) The expression of CENP-H protein was positively correlated with staging and T classification (Table 2). Higher staging and T classification correlated with higher CENP-H expression. In addition, there was a significant difference of CENP-H expression in patients categorized according to gender (*P *= 0.013). The expression of CENP-H protein in male patients was higher than in female patients.

**Table 2:** Correlation between the clinicopathologic features and expression of CENP-H protein

	CENP-H		
		
Characteristics	Low expression	High expression	P
Gender			
Male	67(51.9)	62(48.1)	0.013
Female	31(73.8)	11(26.2)	
Age(y)			
≥ 60	58(56.3)	45(43.7)	0.747
<60	40(58.8)	28(41.2)	
Stage			
I	9(90.0)	1(10.0)	
II a	46(61.3)	29(38.7)	
II b	9 (64.3)	5(35.7)	0.023*
III	30(47.6)	33(52.4)	
IV	4(44.4)	5(55.6)	
Histological differentiation			
Well	30(54.5)	25(45.5)	
Moderate	42(58.3)	30(41.7)	0.637
Poor	26(59.1)	18(40.9)	
Tumor diameter			
≥ 40 mm	37(51.4)	35(48.6)	0.184
<40 mm	61(61.6)	38(38.4)	
Depth of invasion			
Submucosa	8(66.7)	4(33.3)	0.212
Muscularis propria	36(60.7)	22(39.3)	
Adventitia	54(53.8)	47(46.2)	
pT classification			
T1~T2	41(69.5)	18(30.5)	0.019
T3~T4	57(50.9)	55(49.1)	
pN classification			
YES	38(49.4)	39(50.6)	0.057
NO	60(63.8)	34(36.2)	
pMetastasis			
YES	4(44.4)	5 (55.6)	0.426
NO	94(58.0)	68 (42.0)	

*Stage I-II vs. III-IV

### Survival analysis

**Figure 4:**
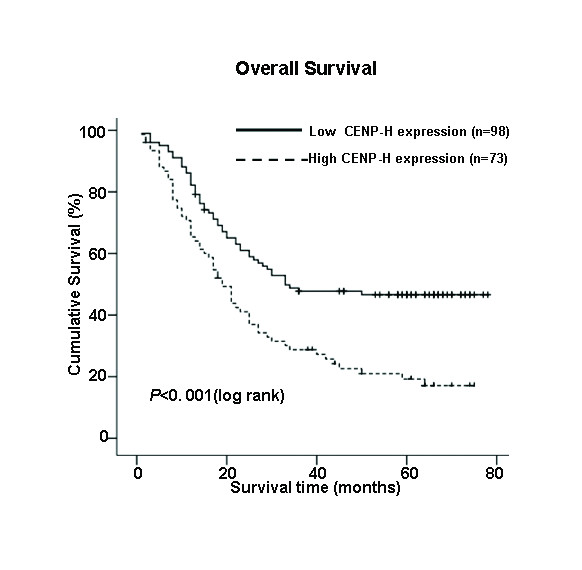
**Kaplan-Meier curves with univariate analyses (log-rank) for patients with low CENP-H expression (bold line) versus high CENP-H expressing tumors (dotted line)**. The median survival of patients with high CENP-H expression was much shorter (19 months) than those with low CENP-H expression (33 months) (P < 0.001, Log-rank).

Kaplan-Meier analysis and the log-rank test were used to calculate the effect of classic clinicopathological characteristics (including gender, stage, N classification) and CENP-H expression on survival. The expression level of CENP-H protein in esophageal carcinoma was significantly correlated with patients' survival time (*P *< 0.001), indicating that higher levels of CENP-H expression was correlated with shorter survival time. The low CENP-H expression group had better survival, whereas the high CENP-H expression group had shorter survival (Fig. 4). The median survival of patients with high CENP-H expression was much shorter (19 months) than those with low CENP-H expression (33 months) (*P *< 0.001, Log-rank).

In addition, N classification, stage and gender were also significantly correlated with survival in Kaplan-Meier analysis and log-rank test (for N classification, *P *< 0.001; for stage, *P *= 0.039 and for gender, *P *= 0.005). We did multivariate survival analysis, which included CENP-H expression level, stage, N classification and gender, to determine if CENP-H expression level is an independent prognostic factor of outcomes. In this analysis, N classification and CENP-H expression were recognized as independent prognostic factors (Table 3). Thus, our findings indicate that CENP-H protein expression level has a significant correlation with prognosis of esophageal carcinoma.

**Table 3:** Univariate and multivariate analysis of different prognostic parameters in patients with esophageal carcinoma by Cox-regression analysis

	Univariate analysis		Multivariate analysis			
		
	No. patients	p	Regression coefficient(SE)	p	Relative risk	95% confidence interval
pN metastasis			0.592(0.197)	0.003	1.807	1.227~2.662
Yes	77	<0.001				
No	94					
Stage		0.039	-0.403 (0.293)	0.169	0.669	0.377~1.187
I-II	99					
III-IV	72					
Gender			-0.496(0.259)	0.056	0.609	0.366~1.012
Male	129	0.005				
Female	42					
CENP-H			0.529(0.200)	0.008	1.698	1.147~2.513
Low expression	98	<0.001				
High expression	73					

**Figure 5:**
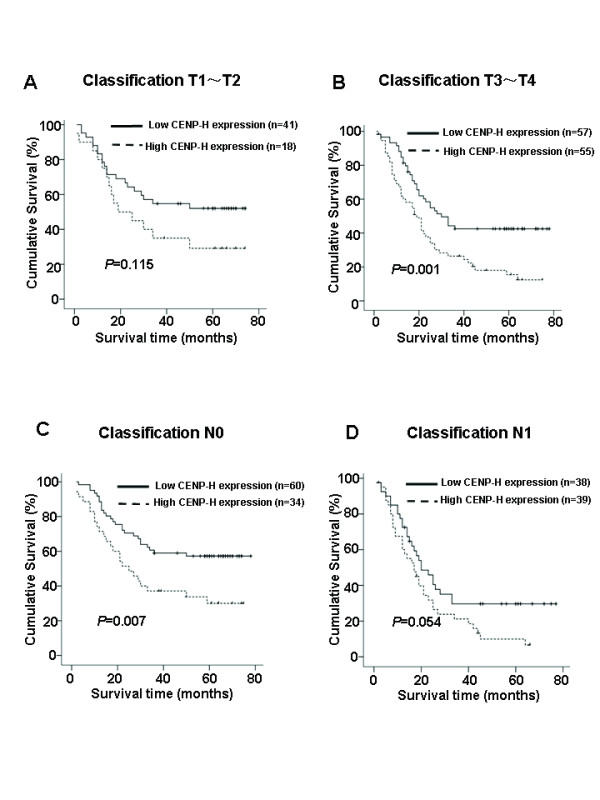
**Kaplan-Meier analysis showing the overall survival of esophageal carcinoma patients categorized according to the T or N classification and status of CENP-H expression**. The statistical significance of the difference between curves of CENP-H high-expressing and low-expressing patients was compared in T1–T2 (A) and T3–T4 (B) patient subgroups. The same analysis was compared in N0(C) and N1(D). P values were calculated by the log-rank test.

We also analyzed the prognostic value of CENP-H expression in selective patient subgroups stratified according to the stage, T and N classification, respectively. Patients with tumors exhibiting high CENP-H expression had significantly shorter overall survival compared with patients with low expression of CENP-H in the T3–T4 subgroup (n = 112; log-rank, *P *= 0.001; Fig. 5B) and the N0 subgroup (n = 94; log-rank, *P *= 0.007; Fig. 5C). A similar analysis of the T1–T2 subgroups (n = 59; log-rank, *P *= 0.115; Fig. 5A) and the N1 subgroup (n = 77; log-rank, *P *= 0.054; Fig. 5D) did not show statistically significant differences between patients with low or high levels of CENP-H expression.

## Discussion

In this report, we presented the first evidence that a kinetochore protein, CENP-H, was overexpressed at both mRNA and protein levels in immortalized cells, ESCC cell lines and most esophageal carcinoma tissues. Overexpression of CENP-H protein was also frequently observed in ESCC specimens and correlated with several aspects of tumor progression summarized in the tumor-node-metastasis classification.

The expression of CENP-H in various kinds of malignant tumors has been reported [14]. However, only a few reports, including our previous article, showed the prognostic relevance of CENP-H expression in neoplasms. Tomonaga et al. [15] first reported that up-regulation of CENP-H was occurred in primary human colorectal cancer tissues as well as in CIN tumor cells. Shigeishi et al. [16] reported that the expression level of CENP-H mRNA was significantly higher in oral squamous cell carcinomas than normal gingivae and found a significant association between the level of expression of CENP-H mRNA and clinical stage in oral squamous cell carcinomas. Our previous study [17] showed that the expression level of CENP-H was higher in nasopharyngeal carcinoma cell lines and in immortalized nasopharyngeal epithelial cells than in the normal nasopharyngeal epithelial cell line at both transcriptional and translational levels. Importantly, patients with higher CENP-H expression had shorter overall survival time, whereas patients with lower CENP-H expression had better survival, and CENP-H expression was an independent prognostic factor. These results indicated an important role for CENP-H in the development and progression of ESCC.

More and more kinetochore proteins have been shown to be associated with carcinogenesis. CENP-F and INCENP are upregulated in human cancer cells [28,29]. CENP-A has been shown to be overexpressed in colorectal cancer cells and is mistargeted to noncentromeric regions[30]. CENP-F has been implicated in malignancy [31,32], and its expression is correlated with tumor size in node-negative breast cancer [33]. The CENP-F gene is amplified and overexpressed in head and neck squamous cell carcinoma [34]. A recent study has shown that increased CENP-F protein levels influence tumorigenesis at early stages of tumor development [35]. These findings suggest that kinetochore proteins up-regulation is a common abnormality in tumors.

Being consistent with our previous study, we found that CENP-H was overexpressed in immortalized and ESCC cell lines as well as in ESCC tissues both at transcriptional and translational levels. We further analyzed the relationship between the expression of CENP-H and clinical characteristics of the patients. There was no significant correlation between the expression of CENP-H and age, histologic classification, histological differentiation, tumor diameter, depth of invasion, pN classification or distant metastasis of esophageal cancer patients. However, there was a significant relationship of CENP-H expression in patients categorized according to stage (*P *= 0.023) and T classification (*P *= 0.019), strongly suggesting that CENP-H can be used as a marker to identify subsets of ESCC cancer patients with more aggressive disease. In addition, our study suggested that the expression of CENP-H protein in male patients was higher than in female patients. Generally, The female patients had better survival, whereas the male patients had shorter survival [39]. Our analysis showed the same result (data not shown), in accordance with the relation analysis between the expression of CENP-H protein and the overall survival (Fig. 4). With regard to the correlation between immunohistochemical CENP-H staining and the prognosis of esophageal carcinoma, we have shown in univariate and multivariate analyses that high expression of CENP-H is an independent prognosticator for patient survival of esophageal cancer. We also analyzed higher CENP-H expression had significantly shorter overall survival during the T3~T4 patients subgroups. The pathological TNM classification indicated that T classification was based on the depth of invasion for carcinogenesis[20]. At the same time, more and more kinetochore proteins have been shown to be associated with carcinogenesis, including CENP-A, CENP-F and INCENP. Shigeishi et al. [16,31] reported CENP-H and CENP-F was closely linked to the increased or abnormal cell proliferation in malignant conditions. It suggests that overexpression of CENP-H might be correlated with abnormal cell proliferation in ESCC. In addition, we found that CENP-H might function as a new prognostic marker in ESCC for the N0 patient subgroups, because in these subgroups there is also a trend toward shorter overall survival times of patients with high expression of CENP-H. The combination of pTNM classification and CENP-H expression level in tumor cells is useful for predicting the prognosis of patients with ESCC. Further studies are clearly needed to verify these findings to establish CENP-H as a prognostic marker in ESCC and to clarify its role in carcinogenesis by functional analysis.

These observations highlight the important role of CENP-H in the development and progression of ESCC. As study in colorectal cancer cell lines showed that overexpression of CENP-H remarkably induced aneupoidy. Moreover, CENP-H stable transfectant of mouse embryonic fibrolast/3T3 cell lines showed aberrant interphase micronuclei, characteristic of chromosomes missegregation [15]. Other reports show that deletion of CENP-H results in an accumulation of cells in metaphase and subsequent cell death as a result of chromosome aberrations and missegregation [38]. CENP-H is a component of active centromere-kinetochore complexes in mammals, colocalizing with both CENP-A and CENP-C, which are found in the inner kinetochore plate throughout the cell cycle [13,36,37]. Inappropriate expression of CENP-H might deplete other centromere-kinetochore components and disrupt the kinetochore complex, or prevent normal kinetochore assembly and consequently cause aneuploidy and induce the development of cancer [22]. These results suggest that CENP-H may be crucial for the appropriate localization and the proper function of the kinetochore. Chromosomal abnormalities, including abnormal chromosome numbers, chromosome deletion, and amplification, are commonly found in ESCC [23-25]. It was reported that defects in mitotic checkpoints occur frequently (~40%) in ESCC cells [26]. Evidence has shown that chromosomal instability plays an important role in the development and progression of ESCC because aneuploidy is frequently found in the earliest stages of tumorigenesis [27]. Up-regulation of CENP-H in esophageal cancer cells may contribute to chromosomal instability and thus play a role in the progression of ESCC.

The development and progression of ESCC stages, including single hyperplasia and single squamous metaplasia, atypical hyperplasia and allotype squamous metaplasia, *in situ *carcinoma, infiltrating carcinoma, and metastatic carcinoma, may involve the accumulation of multiple genetic alterations over a long period of time [40]. Cell immortalization is the ability of normal cells to grow through an in definite number of divisions in culture[41]. Because immortalized cells are capable of unlimited proliferation and represent the early stage of transformation before malignant transformation, we examined CENP-H expression in immortalized esophageal epithelial cell line NE3 and found that CENP-H protein was up-regulated in NE3 cells (Fig. 1). These results suggest that CENP-H may be an early transformation factor of esophageal epithelial cells.

## Conclusion

This is the first study showing the expression of CENP-H in esophageal cancer cell lines as well as tumor tissues, highlighting the clinical significance of CENP-H in esophageal carcinoma. An examination of CENP-H expression is a useful molecular marker for esophageal carcinoma and an indicator for determining malignant properties, including clinical outcome in patients with esophageal carcinoma. However, further studies are needed to clarify the mechanism by which CENP-H is involved in the development and progression of esophageal carcinoma and its exact role in the regulation of chromosome instability in esophageal carcinoma.

## Competing interests

The authors declare that they have no competing interests.

## Authors' contributions

X–ZG and GZ were responsible for data collection and analysis, experiment job, interpretation of the results, and writing the manuscript. L–BS, L–HX were responsible for conducting the data analysis in cooperation with J–YW, W–LL and J–QD. FW, J–YC were responsible for reviewing and scoring the degree of immunostaining of sections. M–SZ was responsible for experimental design, analysis and interpretation. All authors have read and approved the final manuscript.

## Pre-publication history

The pre-publication history for this paper can be accessed here:


